# Glucocorticoids Play a Key Role in Circadian Cell Cycle Rhythms 

**DOI:** 10.1371/journal.pbio.0050078

**Published:** 2007-03-20

**Authors:** Thomas Dickmeis, Kajori Lahiri, Gabriela Nica, Daniela Vallone, Cristina Santoriello, Carl J Neumann, Matthias Hammerschmidt, Nicholas S Foulkes

**Affiliations:** 1 Max-Planck-Institut für Entwicklungsbiologie, Tübingen, Germany; 2 Max-Planck-Institut für Immunbiologie, Freiburg, Germany; 3 European Molecular Biology Laboratory Heidelberg, Heidelberg, Germany; University of Geneva, Switzerland

## Abstract

Clock output pathways play a pivotal role by relaying timing information from the circadian clock to a diversity of physiological systems. Both cell-autonomous and systemic mechanisms have been implicated as clock outputs; however, the relative importance and interplay between these mechanisms are poorly understood. The cell cycle represents a highly conserved regulatory target of the circadian timing system. Previously, we have demonstrated that in zebrafish, the circadian clock has the capacity to generate daily rhythms of S phase by a cell-autonomous mechanism in vitro. Here, by studying a panel of zebrafish mutants, we reveal that the pituitary–adrenal axis also plays an essential role in establishing these rhythms in the whole animal. Mutants with a reduction or a complete absence of corticotrope pituitary cells show attenuated cell-proliferation rhythms, whereas expression of circadian clock genes is not affected. We show that the corticotrope deficiency is associated with reduced cortisol levels, implicating glucocorticoids as a component of a systemic signaling pathway required for circadian cell cycle rhythmicity. Strikingly, high-amplitude rhythms can be rescued by exposing mutant larvae to a tonic concentration of a glucocorticoid agonist. Our work suggests that cell-autonomous clock mechanisms are not sufficient to establish circadian cell cycle rhythms at the whole-animal level. Instead, they act in concert with a systemic signaling environment of which glucocorticoids are an essential part.

## Author Summary

To guarantee normal growth and to avoid tumor formation, the timing of cell division must be under strict control. Remarkably, cells, from bacteria to man, often divide only at certain times of day, suggesting the influence of internal biological clocks. A central pacemaker structure in the brain controls diurnal rhythms of behavior and hormone release. However, biological clocks are also encountered in almost every cell type (so-called “peripheral” clocks), in which they regulate daily changes in cell biology, including cell division. Very little is known to date about how the two clock systems interact. Here, by examining zebrafish strains with defects in hormone production, we find that peripheral clocks require the steroid hormone cortisol to generate daily rhythms of cell proliferation. Interestingly, the daily changes in cortisol levels observed in normal zebrafish are not required to achieve this control; treating the cortisol-deficient strains with constant levels of a drug that mimics the effects of cortisol restores normal cell-division rhythms. Thus, it appears that internal cell timers cooperate with hormonal signals to regulate the timing of cell division.

## Abstract 3

To establish circadian cell cycle rhythms, cell-autonomous clock mechanisms act in concert with a systemic signaling environment of which glucocorticoids are an essential part.

## Introduction

The physiology of most plants and animals changes significantly between day and night. These daily rhythms are generated by endogenous clocks or pacemakers, and persist under constant conditions with a period length of approximately 24 h (hence, they are termed circadian). In vertebrates, cell-autonomous circadian clocks are present in most cell types, and are termed peripheral clocks. In addition, a limited number of specialized central pacemakers such as the suprachiasmatic nucleus (SCN) of the hypothalamus [1,2] appear to play a key role in coordinating the function of peripheral clocks. Although it is known that ocular photoreception synchronizes the SCN pacemaker with the environment, the identity of the pathways that subsequently transmit timing information to the peripheral clocks remains elusive. Current models implicate multiple humoral signals that result indirectly from the SCN circadian control of systemic function, such as feeding behavior [3].

Both cell-autonomous and systemic regulatory mechanisms have been implicated in clock output pathways that relay timing information from the clock to physiological systems. Circadian E box enhancers represent key regulatory elements within the core transcription–translation feedback loop of the vertebrate clock. These promoter elements direct circadian rhythms of transcription of clock genes by acting as binding sites for the clock components CLOCK and BMAL1. Circadian E boxes are also encountered in the promoters of many non-clock genes (so-called clock-controlled genes, e.g., see [4]). Via such target genes and their downstream effectors, peripheral circadian clock components directly regulate many aspects of cell physiology, such as membrane trafficking, detoxification, nutrient metabolism, and the cell cycle [4]. The central SCN pacemaker, in contrast, has been documented to influence systemic functions ranging from locomotor activity rhythms and the sleep–wake cycle to endocrine activity. Thus, the circulating levels of many hormones are under circadian control and so exert their effects only during specific times of day. A major unexplored issue is the relative contribution of cell-autonomous and systemic factors in directing circadian clock outputs. Do certain clock outputs rely solely upon direct peripheral clock regulation, or do they require input from systemic factors, acting either upstream or downstream of the peripheral clocks? Are other outputs driven solely by circadian oscillations of systemic signals?

A particularly interesting clock output is the timing of cell proliferation. Daily rhythms of cell division are conserved across huge evolutionary distances, from cyanobacteria to humans [5,6]. This property has been proposed as a strategy for minimizing the ultraviolet damaging effects of sunlight during critical steps of the cell proliferation. In vertebrates, circadian gating of certain cell cycle steps also occurs in cell lines [7,8]. Furthermore, clock components have been implicated in controlling the transcription of cell cycle regulatory genes [9–12]. These observations imply that the circadian clock may regulate cell cycle progression via cell-autonomous mechanisms. However, given that systemic factors such as hormones are well-known regulators of cell proliferation [13,14], one important question is whether cell-autonomous regulatory mechanisms are sufficient to direct circadian cell cycle rhythms at the whole-animal level.

The zebrafish represents a valuable model for exploring the vertebrate circadian clock and its regulation of cell cycle timing. Robust daily S-phase rhythms are observed in larvae raised under light–dark (LD) cycles [7]. The persistence of these rhythms following transfer of the larvae to constant darkness (DD) conditions demonstrates that they are under control of the circadian clock. Furthermore, consistent with other clock outputs [15], exposure to a LD cycle is essential for the establishment of these rhythms because they are absent in larvae raised in DD. Circadian rhythms of S phase, albeit with lower amplitude, are also observed in zebrafish primary cell lines, implicating cell-autonomous regulation by peripheral clock mechanisms [7]. Interestingly, peripheral clocks in this species can be entrained by direct exposure to LD cycles [16]. However, zebrafish also possess central pacemakers: a structural counterpart of the SCN and a photosensitive pineal complex where nighttime synthesis of the hormone melatonin is directed by an endogenous clock [15,17–20].

Extensive panels of zebrafish mutants that show specific developmental defects in a range of organ systems have been assembled, thanks to large-scale screening efforts [21]. These animals represent potentially powerful tools to dissect the functional contribution of specific organs and tissues to the generation of clock outputs at the whole-animal level. Here, by studying a set of blind mutants, we have demonstrated that ocular photoreception is not required to establish circadian cell cycle rhythms during early larval development. In contrast, a severe attenuation of cell cycle rhythms is observed in mutants that exhibit a reduction or absence of the corticotrope cell lineage in the pituitary gland. Importantly, high-amplitude circadian cell cycle rhythms can be rescued by exposing corticotrope-deficient larvae to tonic concentrations of the glucocorticoid receptor (GR) agonist dexamethasone. Our work reveals the contribution of systemic factors to establishing circadian cell cycle rhythms at the whole-animal level.

## Results

### Ocular Photoreception Is Not Required for Zebrafish Circadian Cell Cycle Rhythms

**Figure 1:**
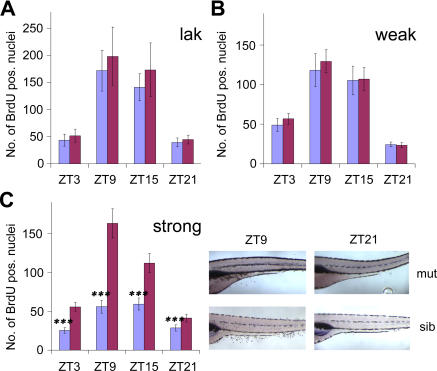
Circadian Cell Cycle Rhythms in Blind Zebrafish Mutants (A) Quantification of BrdU-labeled nuclei in *lak* mutant larvae and wild-type siblings at four circadian time points. The mean number of positive nuclei between swim bladder and anus (*y* axis) was plotted against ZT, hours after lights on, lights off at ZT12) (*x* axis) for mutant (blue) and sibling (red) larvae. Error bars indicate the 95% confidence interval for the mean, and asterisks indicate the statistical significance of differences between mutants and wild-type siblings as measured by the Mann-Whitney-Test. ***, *p* < 0.001. (B) Quantification of BrdU incorporation analogous to (A) for mutants and siblings of the weak *rx3* allele. (C) Left: quantification analogous to (A) for strong *rx3* mutants. Right: representative examples of whole-mount stainings used for the quantification. Mutant (mut) and sibling (sib) larvae at peak (ZT9) and trough (ZT21) points of the cell cycle rhythm are shown. Mutant larvae appear darker, because their melanophores are expanded (lack of visual background adaptation in blind fish). A high-resolution image of this part of the figure is provided as Figure S1. Pooled results from two (A), four (B), and nine (C) independent experiments are shown.

We have previously demonstrated that exposure to a LD cycle is a prerequisite for circadian cell cycle rhythms to be established during early larval development. Whereas zebrafish peripheral clocks are directly light entrainable in vitro, light input through the eyes also plays an important role in entraining the circadian timing system in most vertebrates. We therefore asked whether ocular photoreception might contribute to establishing circadian cell cycle regulation in the zebrafish. We examined cell cycle rhythms in a set of functionally blind mutants using bromodeoxyuridine (BrdU) incorporation as a marker for the S phase of the cell cycle. *lakritz/ath5 (lak)* mutants, which carry a null mutation in a basic Helix-Loop-Helix transcription factor gene, the *atonal homologue 5,* lack the retinal ganglion cell layer [22]. These cells relay light information from the inner retina to the brain. Furthermore, in mammals, the retinal ganglion cells themselves function as a circadian photoreceptor [23]. These mutants are thus particularly well suited for examining the role of ocular photoreception in clock outputs. Mutant and wild-type siblings were raised under a LD regime, and BrdU incorporation was tested at four time points on the sixth day post-fertilization (dpf), before feeding starts. BrdU incorporation rhythms in the *lak* mutant larvae are indistinguishable from their wild-type siblings (Figure 1A) and thus not affected by the absence of ocular light input. To confirm these results, we examined *chokh*/*rx3* mutant fish (carrying mutations in the *retinal homeobox gene 3*), which show a severe impairment of eye and retinal development [24–26]. We analyzed two alleles (“weak,” *chk^t25181^* = *chk^w^,* and “strong,” *chk^t25327^ = chk^s^*) with differing severity of the morphological phenotype [26]. As for the *lak* mutants, in the weak *rx3* larvae, BrdU incorporation rhythms are indistinguishable from the wild-type siblings (Figure 1B). Surprisingly, however, the strong *rx3* mutant larvae show a severe attenuation of the circadian S-phase rhythm compared with their wild-type siblings (amplitude reduced from 4.0-fold to 2.3-fold, Figure 1C and Figure S1). Since both *lak* and weak *rx3* mutants are blind, we conclude that the attenuated S-phase rhythm of the strong *rx3* mutant is functionally unrelated to its blindness. This suggests the presence of additional distinct defects in this mutant.

### *chokh*/*rx3* Mutant Fish Show Normal Clock Gene Expression

**Figure 2:**
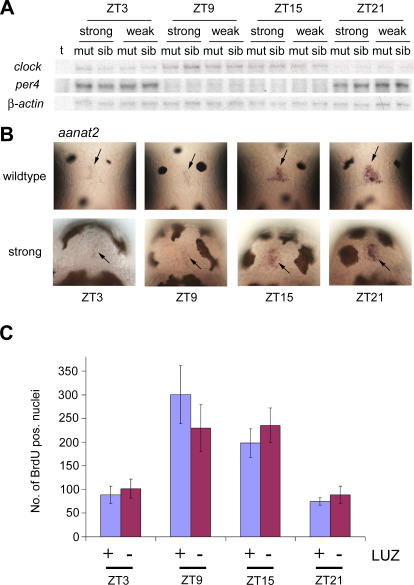
Attenuated Cell Cycle Rhythms Are Not Due to Changes in Circadian Clock Gene Expression or Disturbed Pineal Melatonin Output (A) Circadian expression of clock genes in mutant larvae of both weak and strong alleles of *rx3*. Representative RNase protection assay results for *per4, clock1,* and *β-actin* expression using RNA harvested at the indicated ZTs. “t” represents a tRNA negative control. (B) Circadian expression of *aanat2* in strong *rx3* mutants. Whole-mount in situ hybridization for *aanat2* in strong *rx3* mutants (“strong”) and wild-type siblings (“wildtype”) at four circadian time points (6 dpf). Images show dorsal views of the pineal gland (arrow), rostral up. (C) Quantification of BrdU labeled nuclei in luzindole treated wild-type larvae. Means of BrdU-positive nuclei determined as described in Figure 1 are shown at four circadian time points on 6 dpf for luzindole-treated larvae (+, blue) and control larvae (−, red). Error bars show 95% confidence interval of the mean.

What is the cause of the attenuated cell cycle rhythms in the strong *rx3* mutants? Given the proposed direct link between the cell cycle and the circadian clock [7–10], we first tested whether the cell cycle phenotype was due to a deregulation of the circadian clock itself. We examined mRNA expression of clock genes in larval RNA extracts from wild-type and *rx3* mutant siblings raised in the same conditions as those of the BrdU experiments. We assayed expression of *clock* as a representative of the positive limb [27] and of *per4* for the negative limb [28] of the circadian feedback loop [29]. As shown in Figure 2A, rhythmic expression of *clock* and *per4* in both *rx3* alleles is equivalent to that of their wild-type siblings. Thus, the cell cycle defects in the strong allele cannot be explained by a global deregulation of the circadian clock.

### Melatonin Is Not Essential for Circadian Cell Cycle Rhythms

Could the attenuated cell cycle rhythms of the strong *rx3* mutants be attributed to the disruption of a systemic pathway conveying circadian timing information to the cell cycle? Nocturnal production of the hormone melatonin by the pineal gland is a key central clock output [30]. Recently, melatonin has also been implicated in regulating cell proliferation in larval zebrafish [31]. Pineal mRNA expression of the rate-limiting enzyme of the melatonin synthesis pathway, arylalkylamine N-acetyltransferase (AANAT), is under circadian regulation, with expression high during the night and low during the day ([32] and Figure 2B, upper row). This expression pattern has been used widely as a reliable indicator of the levels of melatonin synthesis in the zebrafish [32–35]. The whole-mount in situ hybridization for *aanat2* in strong *rx3* mutant larvae shown in Figure 2B (lower row) reveals a circadian expression rhythm indistinguishable from wild-type siblings. Thus, the attenuated cell cycle rhythms in strong *rx3* larvae seem unlikely to be explained by defects in this endocrine pathway. As an additional test of the contribution of melatonin, we treated wild-type larvae with luzindole, a specific antagonist of the MT1 and MT2 high-affinity melatonin receptors [31,36]. Cell cycle rhythms were not significantly affected by this treatment, confirming the hypothesis that melatonin production is not required for establishing cell cycle rhythms (Figure 2C). In lower vertebrates, the pineal is a directly photosensitive structure, and light directly affects the production of melatonin [2]. Therefore, our results also indicate that direct pineal photoreception is unlikely to contribute to circadian cell cycle rhythms.

### *chokh*/*rx3* Mutant Fish Show Pituitary Defects

**Figure 3:**
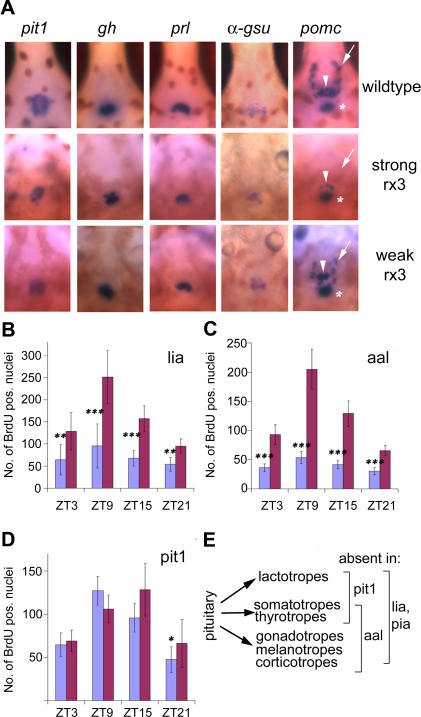
Reduction or Absence of the Pituitary Corticotrope Lineage Results in Attenuated Cell Cycle Rhythms (A) Expression of pituitary markers in *rx3* mutants. Whole-mount in situ hybridizations for *pit1,* growth hormone *(gh),* prolactin *(prl),* the glycoprotein hormone alpha subunit *(α-gsu),* and proopiomelanocortin *(pomc)* (6 dpf), with ventral views through the jaw cartilages, rostral up. Domains of *pomc* expression are indicated by arrows (arcuate nucleus), arrowheads (anterior pituitary), and asterisks (posterior pituitary). strong, strong *rx3* mutants; weak, weak *rx3* mutants; wildtype, wild-type siblings. (B–D) Quantification of BrdU incorporation in the *lia* (B), *aal* (C), and *pit1* (D) mutants. Mean numbers of BrdU-positive nuclei determined as described in Figure 1 are indicated for each time point for mutants (blue) and siblings (red). Error bars show the 95% confidence interval of the mean; asterisks indicate statistical differences between mutant and wild-type values as determined by the Mann-Whitney test: *, *p* < 0.05; **, *p* < 0.01; ***, *p* < 0.001. Pooled results from two (B), five (C), and four (D) independent experiments are shown. (E) Scheme illustrating the pituitary lineages that are lacking (bracketed) in different pituitary mutants: *lia* and *pia* mutants lack all pituitary lineages. *aal* retains the lactotropes and lacks all other lineages. In *pit1,* only gonadotropes and corticotropes/melanotropes are present, and lactotropes, thyrotropes, and somatotropes are absent.

The hypothalamic–pituitary axis is another endocrine pathway with a crucial role in the control of cell proliferation that shows circadian variations of activity [37]. We examined expression of a set of specific pituitary cell-lineage markers in the *rx3* mutants: The transcription factor *pit1* [38], growth hormone, *gh* [39], prolactin, *prl* [39], and glycoprotein hormone alpha subunit, *α-gsu* [38]. Expression of these markers is equivalent in *rx3* mutants of both alleles when compared with their wild-type siblings (Figure 3A). Thus, the somatotrope *(gh),* lactotrope *(prl),* and gonadotrope/thyrotrope *(α-gsu)* lineages appear to be normally formed in the strong *rx3* mutants. However, for the corticotrope/melanotrope lineage marker proopiomelanocortin (*pomc,* [39]), two expression domains show a marked reduction in strong allele *rx3* mutants. The anterior pituitary domain is strongly reduced (arrowhead), and the expression corresponding to the β-endorphin/MSHα synthesizing cells of the arcuate nucleus ([40], arrow) is essentially absent, whereas the posterior pituitary expression domain (asterisk) appears normal. All these domains have a wild-type–like appearance in the weak allele mutant larvae (Figure 3A).

To test whether these differences reflect a general disorganization of the diencephalon, we examined the expression of a number of hypothalamic markers (*somatostatin3*, *isotocin*, and *corticotropin releasing factor;* for details, see Figure S2). The structures labeled by these markers are present in both mutant alleles and show no major disruption, despite the lack of normal eyecups. Thus, the strong *rx3* mutation specifically seems to affect the *pomc*-expressing cells in the anterior pituitary and the arcuate nucleus. Previous studies have established that the anterior pituitary expression domain of *pomc* consists mainly of cells of the corticotrope lineage, whereas the posterior domain also contains melanotrope cells [41]. Furthermore, the arcuate nucleus has been implicated in regulation of the corticotrope axis in mammals [42]. Thus, the reduced number of corticotrope cells in the strong *rx3* mutants might additionally lack normal hypothalamic control. These findings implicate the corticotrope lineage in circadian cell cycle regulation.

### Corticotrope Deficiency Attenuates Cell Cycle Rhythms

Given the pituitary defect in the strong *rx3* mutant, we asked whether disruption of the hypothalamic–pituitary axis would cause similar circadian cell cycle defects to those seen in the strong *rx3* mutants. To address this issue, we examined rhythms of BrdU incorporation in a series of zebrafish mutants that lack either the entire pituitary or specific subsets of pituitary lineages (Figure 3B–3E) [38,40,43–46]. The *fibroblast growth factor 3* mutant *lia/fgf3* (two alleles, [43]) and the proneural basic Helix-Loop-Helix transcription factor *achaete scute-complex like 1a* mutant *pia/ascl1a* [46], which lack the entire pituitary, show severely attenuated rhythms (Figure 3B and unpublished data). Thus, genetic ablation of the pituitary creates a circadian cell cycle phenotype highly similar to that observed for the strong *rx3* mutant. Since *lia* and *pia* mutants show normal *pomc* expression in the arcuate nucleus, we can also exclude a non-pituitary–mediated contribution of β-endorphin/MSHα–expressing arcuate nucleus neurones to cell cycle rhythm generation.

To pinpoint the precise pituitary cell type responsible for the establishment of normal circadian cell cycle rhythms, we examined BrdU incorporation rhythms in two other pituitary mutants that lack subsets of the pituitary lineages (Figure 3E): The protein tyrosine phosphatase *eyes absent 1* mutant *aal/eya1* [44,45], which possesses only the lactotropes, and the POU-domain transcription factor *pit1* mutant [38], in which only the corticotropes/melanotropes and the gonadotropes are present. The *aal* mutants show a similar phenotype to the *lia, pia,* and the strong *rx3* mutants (Figure 3C), demonstrating that the lactotrope lineage alone is not sufficient for establishing circadian cell cycle rhythms. In contrast, the *pit1* mutants are indistinguishable from their wild-type siblings (Figure 3D). Thus, the presence of only the corticotrope/melanotrope and gonadotrope lineages is sufficient to establish wild-type circadian cell cycle rhythmicity. Together with the reduced number of corticotropes observed in the *rx3* mutant embryos, this result strongly suggests that the corticotropes are required for the establishment of the circadian cell cycle rhythms.

### Corticotrope Deficiency Leads to Lowered Larval Cortisol Levels

**Figure 4:**
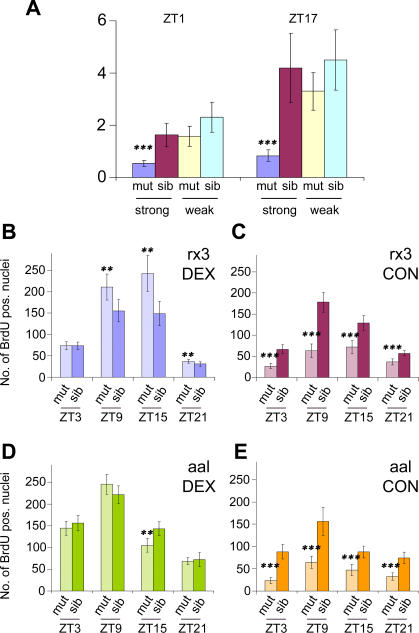
Activation of Glucocorticoid Signaling Rescues Circadian Cell Cycle Defects (A) Cortisol levels in weak and strong *rx3* mutants. Luminescence immunoassay (LIA) of cortisol at two time points for strong allele mutants (blue), strong allele siblings (red), weak allele mutants (orange), and weak allele siblings (green) on 6 dpf. Error bars indicate the 95% confidence interval. Data represent pooled results from three independent experiments. Asterisks indicate statistically significant differences between strong allele mutants and all other larvae as determined by the Mann-Whitney test: ***, *p* < 0.001. (B and C) Effects of dexamethasone treatment on BrdU incorporation in strong *rx3* mutants and wild-type siblings. Larvae were raised from 2 dpf to 6 dpf in the presence (B) or absence (C) of 25 μM dexamethasone (DEX). Means of BrdU-positive nuclei at four circadian time points on 6 dpf are shown for DEX-treated mutants (“mut,” light blue) and wild-type siblings (“sib,” blue) (B), and for untreated (CON) mutants (light red) and wild-type siblings (red) (C). Error bars show the 95% confidence interval of the mean. Asterisks indicate statistical significance of the difference between mutant and wildtype as determined by the Mann-Whitney test: **, *p* < 0.01; ***, *p* < 0.001. Pooled results of four independent experiments are shown. (D and E) Effects of dexamethasone treatment on BrdU incorporation in *aal* mutants and wild-type siblings analogous to (A) and (B). Means of BrdU-positive nuclei are shown for DEX-treated mutants (light green) and wild-type siblings (green) (D) and for untreated mutants (light orange) and wild-type siblings (orange) (E). Pooled results from two independent experiments are shown.

The principal target organ of signaling by the corticotrope axis is the medulla of the adrenal gland (interrenal gland in fish [47]), where it regulates production of glucocorticoids such as cortisol. To explore the mechanism of the cell cycle defect in the strong *rx3* mutants, we measured cortisol levels in 6-d-old mutant and wild-type sibling larvae of both alleles raised under a LD cycle (Figure 4A). All larvae tested show higher cortisol levels at zeitgeber time (ZT)17 than at ZT1. However, mutant larvae of the strong allele have significantly lower levels (*p* < 0.0001) than all other larvae at both time points. Thus, the reduction of corticotrope cells in the strong allele mutant pituitary seems to strongly reduce cortisol levels, pointing to cortisol as a candidate systemic signal required for circadian cell cycle rhythmicity.

### The GR Agonist Dexamethasone Can Rescue the Mutant Cell Cycle Rhythm Phenotype

If cortisol is indeed the systemic signal, it should be possible to rescue circadian cell cycle rhythms by artificially stimulating glucocorticoid signaling in the strong *rx3* mutants. Mutant larvae and wild-type siblings were raised in the presence of the potent glucocorticoid agonist dexamethasone. We then measured BrdU incorporation at four time points on day 6 of development. Strikingly, dexamethasone treatment fully restores high-amplitude circadian cell cycle rhythms in the mutants (Figure 4B). Non-treated control mutant larvae show the typical severely attenuated rhythms (Figure 4C). Similarly, *aal* mutant larvae treated with dexamethasone are indistinguishable from their wild-type siblings (Figure 4D and 4E). In conclusion, tonically activating glucocorticoid signaling during the early days of development can rescue the circadian cell cycle rhythms in cortisol deficient larvae.

Our previous work has shown that the diurnal cell cycle rhythms of zebrafish larvae are under control of the circadian clock, because these rhythms persist upon transfer into constant darkness [7]. We therefore asked whether the rescue effect of dexamethasone treatment could also operate without direct light input. Tonic dexamethasone application could indeed rescue BrdU incorporation rhythms in mutant larvae that were transferred to DD after 5 d of entrainment under a LD cycle (Figure S3), clearly showing that the rescue is due to interaction of glucocorticoids with the circadian clock and not dependent on direct light input.

### Tonic GR Gene Expression

**Figure 5:**
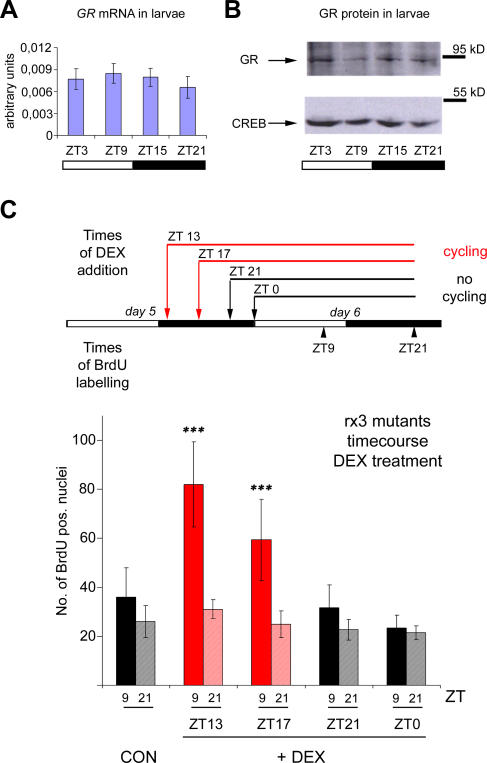
Shorter-Term Treatment with Dexamethasone Also Rescues Cell Cycle Rhythms in Strong *rx3* Mutants (A and B) Expression analysis of the zebrafish GR. (A) GR mRNA expression as determined by quantitative RT-PCR in wild-type larvae at four circadian time points. No significant oscillation is apparent (one-way ANOVA [analysis of variance], *p* = 0.4231). Error bars show the 95% confidence interval of the mean from three independent experiments. As a control, *per4* mRNA levels were assayed in the same samples and shown to exhibit a high-amplitude rhythm (unpublished data; see also Figure2A). (B) Representative Western blot showing expression levels of the GR protein (upper) and of CREB (lower) as a loading control. Consistent with mRNA expression levels, no overt oscillation can be observed. (C) Upper panel: experimental design to define a minimum period of exposure to the tonic dexamethasone (DEX) signal required to rescue cell cycle rhythms in the *rx3* mutants. Black and white bars represent the light and dark periods of day 5 and 6 post-fertilization. Above: times of DEX addition to the medium. Red arrows and lines indicate treatments that are able to increase the cycling amplitude of BrdU incorporation in strong rx3 mutant larvae, whereas black arrows and lines indicate those that fail to do so. Below: times for BrdU labeling (as 15-min pulses) and harvesting on day 6 (indicated as ZT time points). Lower panel: quantification of BrdU incorporation in strong *rx3* mutants following the treatments outlined in the upper panel. Peak (ZT9, dark color) and trough (ZT21, light color) mean values on day 6 are shown for untreated controls (CON) and the four times of DEX addition on day 5 (+DEX). Red bars: treatments that result in a significant difference between peak and trough values (Mann-Whitney-Test; ***, *p* < 0.001), black bars: no significant differences observed between peak and trough values. Error bars indicate the 95% confidence interval of the mean. Data are pooled from six independent experiments.

We wished to explore in more detail how cortisol affects cell cycle rhythmicity. We first tested the hypothesis that the circadian clock might regulate expression levels of the glucocorticoid receptor gene *(GR)* and thereby confer a circadian rhythm of sensitivity to the receptor ligand. Such a mechanism would enable even tonic levels of the ligand to activate GR signaling pathways with a circadian rhythm. Furthermore, recent reports have highlighted that many members of the nuclear receptor superfamily show circadian cycling of transcript levels [48]. We thus prepared a time course of total RNA and protein extracts from wild-type sibling larvae during one LD cycle. Quantitative real-time PCR analysis failed to detect any significant change in GR mRNA levels during the course of the LD cycle (Figure 5A). Consistently, levels of an 82-kDa immunoreactive protein corresponding to the zebrafish GR also did not cycle as determined by Western blotting analysis (Figure 5B). These results indicate that the circadian clock does not simply affect global expression levels of the GR.

### Shorter Dexamethasone Treatments Also Rescue Cell Cycle Rhythms

Many studies have documented the functional complexity of the glucocorticoid signaling pathway in vivo [49]. Lack of cortisol during development and larval growth might generally alter larval physiology and thereby also indirectly affect cell cycle rhythms. Rescue of high-amplitude cell cycle rhythms in *rx3* mutant larvae by continuous exposure to dexamethasone from early development onwards could act via rescuing developmental defects as well as by affecting cells more directly. In order to address this point, we systematically tested the effect of reducing the duration of exposure to dexamethasone on cell cycle rhythms. We supplemented the medium with dexamethasone at progressively later stages before harvesting on day 6 of larval development (Figure 5C and unpublished data). Addition of dexamethasone as late as the night before sampling (day 5) still resulted in a significant increase in cell cycle amplitude. Dexamethasone delivered at later time points failed to rescue the rhythm. Given that all the major organ systems have developed and are functional at this freely feeding larval stage [50], this would exclude a major role for indirect developmental mechanisms.

## Discussion

The zebrafish represents an attractive model to explore how circadian clock outputs are regulated at the whole-animal level. We have previously implicated a contribution of directly light-entrainable peripheral clocks in the cell-autonomous control of circadian rhythms of S phase. Here, by studying panels of zebrafish mutants affecting development of the eye and hypothalamic–pituitary axis, we have been able to define the regulatory contribution of these structures to establishing circadian cell cycle rhythms. This study illustrates the power of using complementary sets of zebrafish mutants in the genetic dissection of physiological pathways in vivo.

We have shown that ocular photoreception is dispensable for establishing this clock output function, potentially reinforcing the notion that cell-autonomous light sensing plays a key role in cell cycle entrainment in the zebrafish. However, in common with most lower vertebrates, zebrafish possess additional extraocular specialized photoreceptor tissues: the pineal complex [2] and also the so-called deep brain photoreceptors that line the third ventricle of the diencephalon [51,52]. Since no zebrafish mutants are available to date that specifically lack these photoreceptors, it is problematic to assess their contribution. We used an alternative pharmacological approach to interfere with the melatonin signal, the major output of the pineal gland, and thereby tested whether photoreception through the pineal complex might affect cell cycle rhythms. Because treatment of larvae with the melatonin receptor antagonist luzindole did not change circadian cell cycle rhythms, pineal light reception is not strictly required for the timing of circadian cell cycle progression. However, it is still conceivable that light input from the pineal conveyed via neuronal pathways may contribute to the timing of the cell cycle [53–55]. Also, one type of dedicated photoreceptor might be able to substitute for lack of input from the other types, leading to functional redundancy of inputs from, e.g., the eye and the pineal complex. Finally, the direct peripheral light reception alone might also be sufficient to time this clock output in the context of the whole animal. Ultimately, mutant zebrafish that lack all specialized photoreceptor cells will be required to assess the relative contribution of directly light-sensing peripheral clocks to entraining the circadian cell cycle.

By studying pituitary mutants with overlapping cell-lineage defects, and by our demonstration that pituitary corticotropes are severely reduced in strong allele *rx3* mutant larvae, we have implicated this pituitary cell lineage in the establishment of circadian cell cycle rhythms. Furthermore, levels of cortisol in strong *rx3* mutants are reduced and, importantly, a GR agonist rescues the circadian cell cycle defects in both strong *rx3* and pituitary mutants. These findings point to glucocorticoids as a requirement for high-amplitude cell cycle rhythms.

Previously, glucocorticoids have been implicated in the entrainment of peripheral circadian clocks in mammals. Injection of dexamethasone into mice can reset the phase of peripheral oscillators [56]. However, mice lacking the GR show normal clock gene expression rhythms in the liver [56]. Furthermore, here we observe normal circadian clock gene cycling in the strong *rx3* mutant larvae (Figure 2A), despite their low levels of cortisol. Consistently, also in the *aal* mutants, *per4* exhibits normal circadian rhythms of expression (Figure S4). Finally, zebrafish cells grown in cortisol-depleted medium still show normal clock expression under a LD cycle (Figure S5B, see below). Thus, glucocorticoid signaling is not an absolute requirement for the regulation of the peripheral pacemakers themselves. Our data directly implicate glucocorticoids in the regulation of clock output.

Glucocorticoids are well known to influence cell proliferation, both in vitro and in vivo. For example, dexamethasone can stimulate myoblast proliferation [57] and enhance the mitogenic response of fibroblasts to epidermal growth factor (EGF) [58], whereas it inhibits cell division in a lymphosarcoma cell line [59]. In addition, at the whole-animal level, corticosteroids have been reported to affect the capacity of the liver to regenerate after hepatectomy in rats [60]. Our results indicate that part of the effect of corticosteroids on cell proliferation might be brought about by cooperation with circadian clock output pathways.

At which level of organization might this interaction occur? Given the wealth of physiological targets of glucocorticoids, they might function through indirect, systemic pathways. For example, in zebrafish larvae, they might be involved in the maturation of organ systems and their physiological functions during development. However, our finding that dexamethasone treatment starting 10 h before sampling still rescues cell cycle rhythms in *rx3* mutants makes a long-term effect on development unlikely. Nevertheless, other indirect systemic pathways might act within this time frame. For example, glucocorticoids could stimulate the release of mitogens from neighboring tissues or influence the release of other hormones (e.g., see [61–63]). Alternatively, they might act directly at the level of the proliferating cells themselves. We tested a potential cell-autonomous role for glucocorticoids in circadian cell cycle rhythms by examining zebrafish cell cultures grown in charcoal-treated (and thereby steroid-depleted) medium (Figure S5). Whereas circadian clock gene expression is normal in these cultures (Figure S5B), circadian cell cycle rhythms are severely attenuated (Figure S5A), thus mimicking the situation in cortisol-deficient larvae. However, in this cell culture system, treatment with dexamethasone does not rescue the attenuation (unpublished data). One explanation for this result could be that glucocorticoids might need to act synergistically with other substances that are also depleted from the medium by charcoal treatment. Taken together, although there are some hints for a direct cell-autonomous action of glucocorticoids on circadian cell cycle rhythms, more indirect systemic effects certainly cannot be excluded. Indeed, given the multifaceted actions of glucocorticoids, confinement of their effects on cell cycle progression to a single level would be rather surprising. In the animal, glucocorticoids might help to create a systemic signaling environment, within which they could also exert more direct effects.

What is the relative importance of systemic and peripheral circadian clock mechanisms in glucocorticoid-mediated circadian cell cycle regulation? Circadian rhythms of circulating glucocorticoids have long been recognized as a clock output in various vertebrates [37]. However, here we show that the role played by glucocorticoids in circadian cell cycle rhythms does not necessarily involve conveying timing information via changes in circulating levels. Rather, a constant glucocorticoid signal can rescue rhythms of cell cycle in cortisol-deficient mutant larvae. In the animal, the timing information might stem from another cycling systemic signal, or it might be provided by peripheral circadian clocks. Circadian changes in glucocorticoid levels might then reinforce the peripheral timing information in cell cycle control, or they might be required for other physiological functions.

**Figure 6:**
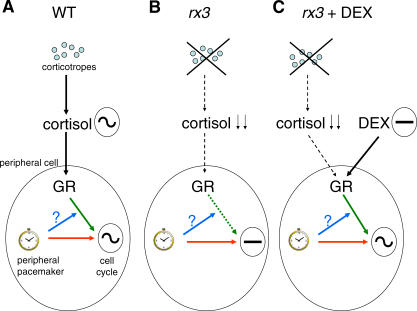
Working Model for Glucocorticoid Action on Circadian Cell Cycle Rhythms (A) In wild-type larvae, corticotrope function is associated with normal cortisol levels (that exhibit circadian cycling). In target cells, cortisol activates GR signaling (green arrow) that may be permissive for the peripheral clock regulation of cell cycle. In addition, cycling cortisol levels may confer circadian rhythms of GR signaling that potentially reinforce cell cycle rhythmicity. The red arrow indicates the peripheral circadian clock control of cell cycle, and the blue arrow symbolizes the potential gating of GR signaling by the peripheral pacemaker. Cooperation between the peripheral clock mechanism and the GR signaling pathway generates a circadian rhythm of cell cycle. (B) In corticotrope-deficient mutants, circulating cortisol levels are significantly reduced. In the absence of normal GR signaling, peripheral clock input is not sufficient to generate cell cycle rhythms. Dashed arrows indicate attenuated pathways. (C) Cell cycle rhythms are rescued in corticotrope-deficient mutants by tonic dexamethasone (DEX) treatment. Thus timing information is provided by peripheral circadian clocks and is not reliant on cycling cortisol levels.

How can a constant glucocorticoid signal lead to a rhythmic output? We propose a working model in which a certain level of glucocorticoid signaling may be permissive (Figure 6A, green arrow) for peripheral circadian clock regulation of genes involved in cell proliferation (Figure 6A, red arrow). Alternatively, the clock could gate responsiveness to the glucocorticoid signal, which would then act to regulate cell proliferation (Figure 6A, blue arrow). In either scenario, in corticotrope-deficient mutants, GR signaling is attenuated, and then peripheral clock input alone would be insufficient to generate full cell cycle rhythms (Figure 6B). Furthermore, in the rescue experiments, tonic dexamethasone treatment activates GR signaling and would restore cell cycle rhythms in the mutants with timing information being provided by the peripheral circadian clock (Figure 6C). In this model, cycling cortisol levels are not required for, but they might reinforce cell cycle rhythmicity.

An attractive mechanism for gating responsiveness to glucocorticoids would involve circadian clock regulation of expression of the GR itself. Expression of many nuclear receptor transcripts is under circadian clock control in different peripheral tissues [48]. However, here we show that the levels of the GR transcript as well as the protein are not subject to significant day–night variation. Thus, a simple scenario in which transcription of the *GR* gene is a regulatory target of peripheral clock components, e.g., via E box elements, appears unlikely. One can speculate that if indeed the receptor is under clock control, then this may occur at the post-translational level. Alternatively, circadian clock control might operate at various levels on other elements of the glucocorticoid signaling pathway, perhaps downstream of the receptor itself [64,65].

Glucocorticoids can increase the amplitude of cell cycle rhythms in strong *rx3* mutants even when they are delivered the night before assaying BrdU incorporation. Specifically, they have to be present at least before ZT17, or 16 h before the peak of BrdU incorporation at ZT9 is reached. Adding dexamethasone at ZT21 (12 h before the expected peak) is not sufficient. This might reflect either a minimum time needed to exert downstream effects on the cell cycle, or a requirement for the presence of glucocorticoids at a certain time of day. Interestingly, the last time point with rescue capability coincides with the natural peak in cortisol levels in wild-type larvae (Figure 4A). Experiments involving a precisely controlled temporal activation and inactivation of the glucocorticoid signaling pathway will be needed to decide between these possibilities.

In summary, our results call for a re-evaluation of existing models that account for control of circadian cell cycle timing purely via the direct regulation of gene expression by peripheral clock components. We reveal a requirement for endocrine regulation involving glucocorticoids that operates downstream of the clock mechanism itself. It is tempting to speculate that many other clock outputs may involve similar contributions from cell-autonomous and systemic control elements.

## Materials and Methods

### Fish care, RNase protection assay, BrdU labeling, and dexamethasone treatment.

Fish were raised and bred according to standard procedures [50]. RNase protection analysis was carried out as described previously [27], and the *per4, clock1,* and *β-actin* probes have been described [27,28,66]. The raising of larvae under controlled lighting and temperature conditions, and the BrdU labeling procedure have been described in [7]. Briefly, ten mutant and ten wild-type sibling larvae each were sorted into cell culture flasks on day 2 post-fertilization and raised at 25 °C under a 12-h light:12-h dark cycle until day 6 of development. Three hours after “lights on” (ZT = 3), the larvae of one flask were incubated for 20 min with BrdU before fixation in 4% paraformaldehyde/PBS, and this procedure was repeated at three additional time points at 6-h intervals. The cell culture BrdU incorporation experiments (AB.9 cells, ATCC) were carried out as described in [7]. Cells were raised in L15 medium supplemented with gentamycin, streptomycin, penicillin [16], and either 15% fetal bovine serum (FBS) or 15% charcoal-treated FBS (both Biochrom, Berlin, Germany).

The pituitary and *lak* mutants are not morphologically distinguishable from wild-type siblings early in development. Thus, two flasks with 25 larvae each from a cross of heterozygous carriers were sorted and processed as described above to ensure the presence of a sufficient number of mutants per time point. *lak* mutant larvae can be recognized by their expanded melanophore phenotype later in development. To identify the pituitary mutants, larvae were stained for growth hormone expression by whole-mount in situ hybridization (see below) and then stained for BrdU incorporation.

Dexamethasone treatment was carried out essentially as described by [41]. Dexamethasone (Sigma, St. Louis, Missouri, United States) was dissolved in distilled water at 1 mM as a stock solution, then diluted further in E3 medium to a final concentration of 25 μM. Ten strong allele *rx3* mutants and wild-type siblings were sorted into each cell culture flask on day 2 of development and raised in the presence or absence of dexamethasone until BrdU labeling on day 6 as described above. Luzindole treatments were performed similarly, with a stock solution of 0.01 M luzindole (Sigma) in ethanol diluted further in E3 to a final concentration of 0.00001 M [31]. Twenty larvae were raised in 25 ml of E3 at this luzindole concentration from day 2 of development, and on day 4, an additional 30 μl of luzindole stock solution were added to compensate for potential degradation of the compound. Control larvae were treated with equivalent solvent (ethanol) concentrations only.

### In situ hybridization.

In situ hybridization was carried out essentially as described [50], with the following modifications: The 4% paraformaldehyde fixation step after rehydration was omitted. Larvae were washed twice in PBS+0.1% Tween-20 (PBST), then rinsed for 2 min in distilled water, incubated for 7 min in pre-cooled acetone at −20 °C, passed through distilled water for 2 min at room temperature, and washed 3×5 min in PBST. Then, larvae were digested with 1 mg/ml of collagenase P (Roche, Basel, Switzerland) in PBS with 1% BSA and 1% DMSO at room temperature for 45 min, before fixation for 20 min at 4 °C in 4% paraformaldehyde. After five washes with PBST, larvae were prehybridized with HYB buffer for 4–6 h, then incubated with the probes overnight. After antibody incubation, larvae were washed 6 × 15 min in PBST, then staining was developed for several hours at room temperature and up to overnight at 4 °C. To remove pigmentation, larvae were incubated in 3 ml of 10% H_2_O_2_/methanol overnight, followed by addition of 12 ml of PBST, incubation overnight, and several washes with PBST. Probes used were *aanat2* [32], *pit1* [38], *gh* [39], *prl* [39], *α-gsu* [38]*, pomc* [38], *isotocin* [67], *ppss3* [68], and *per4* [28]. *corticotrophin relasing factor* (*crf* ) was cloned in our laboratory.

### Quantitative RT-PCR.

Total RNA was extracted from triplicate samples using Trizol RNA isolation reagent (GIBCO-BRL, San Diego, California, United States) according to the manufacturer's instructions. The RNA (3 μg) was reverse-transcribed using Oligo(dT) primer (Amersham Biosciences, Little Chalfont, United Kingdom) and SuperScript III reverse transcriptase (Invitrogen, Carlsbad, California, United States). *per4* mRNA levels were determined by real-time PCR using the DNA Engine Opticon thermocycler (Bio-Rad, Hercules, California, United States) following the manufacturer's instructions. First-strand cDNA aliquots from each sample were diluted 20× and served as templates in a PCR consisting of master mix, SYBR Green I fluorescent dye (Bio-Rad), and 400 nM gene-specific primers. Copy numbers were normalized using *β-actin* controls. Primer sequences were *per4:* 5′-CCGTCAGTTTCGCTTTTCTC-3′ and 5′-ATGTGCAGGCTGTAGATCCC-3′; *glucocorticoid receptor:* 5′-CGGACAGAGCTTCCTCTTTG-3′ and 5′-CTGCTGCATTCCACTGACAT-3′; and *β-actin:* 5′-TCCTGCTTGCTAATCCAC-3′ and 5′-ACCACCTTCAACTCCATC-3′.

### Western blotting.

Protein extracts were prepared by homogenizing 20 larvae per time point in 100 μl of Laemmli buffer. A total of 10 μl of the homogenate was loaded on a 6% SDS polyacrylamide gel, then Western blotting (BioRad) was carried out using an anti-human glucocorticoid receptor α (sc-1002; Santa Cruz Biotechnology, Santa Cruz, California, United States) and anti-mouse CREB antibodies (Upstate Biotechnology, Billerica, Massachusetts, United States), and visualized with the ECL detection system (Amersham Biosciences).

### Cortisol luminescence immunoassay.

Twenty-five larvae each were raised in cell culture flasks with 20 ml of E3 medium under a LD cycle and a constant temperature of 25.3 °C. At each time point, larvae were rapidly transferred to 2-ml Eppendorf tubes, the medium was removed, and the larvae were snap frozen in liquid nitrogen and stored at −80 °C until further processing. For each time point and condition, three identical flasks were used per experiment. Larvae were homogenized on ice with a microgrinder (Eppendorf, Hamburg, Germany), then extracted with 500 μl of cold ethanol. After centrifugation for 10 min at 3,000 rpm at 4 °C, the supernatant was recovered and evaporated in a SpeedVac. The resulting pellet was resuspended in 20 μl of standard A buffer of the IBL cortisol LIA kit (IBL, Hamburg, Germany) and measured following the instructions provided by the supplier. The LIA plate was read by a VICTOR light 1420 luminometer (Wallac/Perkin Elmer, Wellesley, Massachusetts, United States), and the raw data were analyzed using the MikroWin2000 program, version 4.23 (Mikrotek Laborsysteme, Overath, Germany).

### Statistical analysis.

Statistical analysis was performed using the GraphPad Prism version 4.00 for Windows (Graph Pad Software, http://www.graphpad.com).

## Supporting Information

Figure S1 High-Resolution View of Representative Whole-Mount BrdU Stainings Shown in Figure 1
Mutant (mut) and sibling (sib) larvae at peak (ZT9) and trough (ZT21) points of the cell cycle rhythm are shown.
(8.0 MB TIF)

Figure S2 Normal Expression of Hypothalamic Markers in *rx3* Mutants Whole-mount in situ hybridization with probes against *somatostatin3 (ppss3)* [68] (A), *isotocin (itnp)* [67] (B), and *corticotropin releasing factor (crf)* (C) of strong and weak *rx3* mutants and wild-type siblings on 6 dpf. For each panel: wild-type (“wildtype”), strong mutant (“strong”), and weak mutant (“weak”); top row shows a lateral view with the anterior end left (in wild-type: left eye removed to allow a better view of the brain stainings), and the bottom row shows a dorsal view with the anterior end left. Expression patterns of these representative probes appear normal, despite the lack of normal eyecups in the mutants.
(6.4 MB TIF)

Figure S3 Dexamethasone Interacts with the Circadian Clock to Rescue Circadian Cell Cycle Defects Effects of dexamethasone treatment on BrdU incorporation in strong *rx3* mutants and wild-type siblings after transfer into constant darkness. Larvae were raised from 2 dpf to 6 dpf in the presence (A) or absence (B) of 25 μM dexamethasone (DEX). Lights were turned off on day 5 at ZT12, and BrdU pulses and harvesting carried out at circadian time (CT) 3, 9, 15, and 21 on the following day. Means of BrdU-positive nuclei at four circadian time points on 6 dpf are shown for DEX-treated mutants (“mut,” blue) and wild-type siblings (“sib,” red) (A), and for untreated (CON) mutants (blue) and wild-type siblings (red) (B). Untreated mutants do not show significant circadian cycling under these conditions (Kruskal-Wallis test, *p* = 0.3022). Error bars show the 95% confidence interval of the mean. Asterisks indicate statistical significance of the difference between mutant and wild-type as determined by the Mann-Whitney test: **, *p* < 0.01; ***, *p* < 0.001. Pooled results of two independent experiments are shown.
(3.1 MB TIF)

Figure S4 Normal Expression of *per4* in *aal* Mutants Whole-mount in situ hybridization with probes against *per4* of *aal* mutants and wild-type siblings. Mutants and wild-type siblings were discriminated by co-hybridizing with a probe against *growth hormone* (unpublished data).
(A–D) Overview of staining results for mutants (C and D) and wild-type siblings (A and B) at ZT 12 (A and C) and 23 (B and D).
(E and F) Close-ups of mutant and wild-type sibling larvae at ZT12 (E) and ZT23 (F). Strong *per4* staining is visible in both mutant and wild-type siblings at ZT23, whereas barely any staining can be detected in larvae of both genotypes at ZT12.
(5.5 MB TIF)

Figure S5 Low Cortisol Levels Attenuate Circadian Cell Cycle Rhythms in Cultured Cells Downstream of the Circadian Clock (A) BrdU incorporation in AB9 zebrafish cell culture cells raised in charcoal-treated medium (a standard procedure to selectively reduce steroid levels in culture medium [69–71], −GCs) and normal medium (+GCs). For each time point, the mean OD_450_ (optical density) measurement of the BrdU enzyme-linked immunosorbent assay (ELISA) from eight independent wells plus the 95% confidence interval of the mean are shown for cells raised in charcoal-treated (blue) or normal serum (red). One representative experiment is shown. Removal of glucocorticoids results in severe attenuation of circadian cell cycle rhythms.
(B) Clock gene expression in AB9 cells raised in charcoal-treated and normal medium. Quantitative RT-PCR results for *per4* expression are shown. Cells were grown in 25-cm^2^ culture flasks for 5 d under a LD cycle before RNA isolation. Experiments were carried out in triplicate. Error bars indicate the 95% confidence interval of the mean. Clock gene expression is indistinguishable between cells raised in glucocorticoid-depleted medium and those raised in normal medium, indicating that glucocorticoids exert their effects on the cell cycle downstream of the circadian clock.
(973 KB TIF)

### Accession Numbers

The GenBank accession numbers (http://www.ncbi.nlm.nih.gov/Genbank) for the genes discussed in this paper are *α-gsu* (NM_205687), *aal/eya1* (NM_131193), *aanat2* (NM_131411), *atonal homologue 5* (AB049457), *clock* (NM_130957), *corticotropin releasing factor* (DQ250674), *glucocorticoid receptor* (NM_001020711), *lia/fgf3* (NM_131291), *isotocin* (AY069956), *per4* (NM_212439), *pia/ascl1a* (NM_131219), *pit1* (AY421970), *pomc* (NM_181438), *prl* (NM_181437), *retinal homeobox gene 3* (NM_131227), and *somatostatin3* (BI472739 and BI473045).

## Abbreviations

BrdU: bromodeoxyuridine
CT: circadian time
DD: constant dark
dpf: day post-fertilization
GR: glucocorticoid receptor
LD: light–dark
RPA: RNAse protection assay
SCN: suprachiasmatic nucleus
ZT: zeitgeber time
